# Health care costs of influenza-related episodes in high income countries: A systematic review

**DOI:** 10.1371/journal.pone.0202787

**Published:** 2018-09-07

**Authors:** Carlo Federici, Marianna Cavazza, Francesco Costa, Claudio Jommi

**Affiliations:** 1 Cergas (Centre for Research on Health and Social Care Management), SDA Bocconi School of Management, Milan, Italy; 2 Department of Pharmaceutical Sciences, Università del Piemonte Orientale, Novara, Italy; Rijksuniversiteit Groningen, NETHERLANDS

## Abstract

**Introduction:**

This study systematically reviews costing studies of seasonal influenza-like illness (ILI) in high-income countries. Existing reviews on the economic impact of ILI do not report information on drug consumption and its costs, nor do they provide data on the overall cost per episode.

**Methods:**

The PRISMA-P checklist was used to design the research protocol. Studies included were cost of illness analysis (COI) and modeling studies that estimated the cost of ILI episodes. Records were searched from January 2000 to December 2016 in electronic bibliographic databases including Medline, Embase, Science Direct, the Cochrane Library, the Centre for Reviews and Disseminations of the University of York, and Google scholar. References from the included studies were hand-searched for completion. Abstract screening, full-text analysis and data extraction were performed by two reviewers independently and discrepancies were resolved by discussion with a third reviewer. A standardized, pre-piloted form was used for data extraction. All costs were converted to 2015 US$ Purchasing Power Parities.

**Results:**

The literature search identified 5,104 records. After abstract and title screening, 76 studies were analyzed full-text and 27 studies were finally included in the review. Full estimates of the cost per episode range from US$19 in Korea to US$323 in Germany. Particularly, the cost per episode of laboratory confirmed influenza cases was estimated between US$64 and US$73. Inpatient and outpatient services account for the majority of the costs. Differences in the estimates may reflect country-specific characteristics, as well as other study-specific features including study design, identification strategy of ILI cases, study populations and types of costs included in the analysis. Children usually register higher costs, whereas evidence for the elderly is less conclusive. Patients risk-profile, co-morbidities and complications are the other important cost-drivers. None of the papers considered appropriateness in resource use (e.g. abuse of antibiotics). Despite cost of illness studies have ultimately a descriptive role, evidence on (in)appropriateness is useful for policy-makers.

## Introduction

Influenza is defined as an acute viral infection of the respiratory tract, with symptoms marked by inflammation of the nasal mucosa, the pharynx, and conjunctiva, and by headache and severe, often generalized, myalgia. It usually occurs in winter months with epidemic outbreaks occurring every year, causing substantial morbidity and even mortality when followed by severe clinical complications. The World Health Organization (WHO) estimates that worldwide, these annual epidemics result in about 3 to 5 million severe cases of illness and about 250,000 to 500,000 deaths [1].

Given the high annual morbidity, influenza episodes put also a considerable strain on health care systems, with expenditures originating from both inpatient and outpatient settings. Previous research has estimated that in 2003, direct medical costs for the treatment of influenza were about $10.4 billion in the United States [2], whereas in Italy, Lai et al. reported a cost for seasonal influenza epidemics (1999–2008) ranging between US$0.3 and US$2.7 billion per year, with an annual average of US$1.4 billion [3]. Despite being expensive, hospitalization events are rare, while outpatient medical services and drugs are deemed to represent a considerable share of total health-care spending. For example, Molinari et al. [2] estimated that outpatients visits in the United States account for about 30% of total influenza costs.

In addition, management of influenza-like symptoms might encompass potentially inappropriate expenditures including unnecessary emergency visits, or inappropriate pharmacological management [4]. For example, the problem of antivirals, such as oseltamivir, being prescribed to patients who do not actually have influenza is widely acknowledged among the medical community. Conversely, inappropriate prescriptions of antibiotics to laboratory-confirmed influenza patients has been reported to be as high as 30% in five US centers [5]. Not only such practices prompt unnecessary costs to the health-care system and longer time to recovery for patients, but ultimately, they may also favor the emergency of drug-resistant infections in the long term.

Furthermore, the burden of influenza extends to the wider societal perspective, including direct health-related out-of-pocket expenditures (OOPs), and indirect costs that are mainly driven by productivity losses due to work absenteeism, and informal caregivers time.

Lastly, both clinical outcomes and costs vary by population sub-groups, with higher risk patients accounting for higher costs due to the insurgence of complications and the need of more specific treatment strategies. Particularly vulnerable groups are pregnant women, children, the elderly, and individuals with specific chronic medical conditions, such as chronic heart diseases, lung diseases, or HIV/AIDS.

Previous reviews have analyzed the economic burden of influenza on both direct healthcare costs and indirect societal costs [6–8]. For example Peasah et al. reports nationally-aggregated and per capita costs of hospitalization events, outpatient services and productivity losses [8], whereas Dao et al. collected data on direct medical and non-medical costs as well as indirect costs [6]. These previous works generally provide aggregate estimates per macro-categories of costs, per capita costs and average cost per item. For example, Peasah et al. report that indirect costs account for more than 50% of total costs in five out of eight studies reviewed, and that total costs per capita range between US$ 1 (Thailand) to US$ 63 (USA). The authors also provide separated cost estimates for hospitalization events, outpatient visits and per-day productivity losses [8]. However, they do not provide any data on the costs per episode, nor they report specific cost items and drug expenditures, or any consideration on inappropriate spending for the management of influenza.

The main objective of this review is to update the current literature with more detailed data on the main determinants of direct health-care expenditures for influenza. To this end, the primary aims are to i) collect and analyse detailed data on influenza-driven health-care costs per episode, with a specific focus on inpatient and outpatient costs, emergency department (ED) services, and drugs; ii) analyse the available evidence about drug expenditures, particularly symptomatic treatments and over-the-counter (OTC) drugs, and iii) report on how different cost items vary by population subgroups such as specific age and risk profiles.

Secondary aim of the proposed review is to provide evidence, if any, about inappropriate influenza-related use of direct healthcare resources (i.e. inappropriate prescriptions of drugs, or unnecessary ED visits).

## Methods

A research protocol for this review was drafted using the PRISMA-P checklist [9]. The types of studies considered were all types of cost of illness analyses (COI), including both incidence and prevalence-based analyses, using either prospective or retrospective data. Modeling studies that empirically estimated the cost per influenza-related episode and then extrapolated it to a wider population were included as well.

Since this review was not aiming to estimate the impact of specific interventions (e.g. the impact of vaccines, or rapid influenza diagnostic tests on costs or cost-effectiveness), all cost-effectiveness and budget impact analyses, or other types of comparative studies were not included. Costs may be estimated through either a bottom up or top down approach, with data sources including administrative data, medical records or patient surveys. Other types of studies and data sources, not specifically covered by the defined inclusion criteria, were assessed on a case-by-case basis, and included if deemed informative to the aim of the review.

Participants of the studies were from the general population living in high income countries according to the list provided by the World Bank [10]. This restriction in scope is justified by the fact that cost estimates in low and middle-income countries are poorly comparable to those in high income countries, mainly because of differences in the healthcare systems, including funding schemes, share of private co-payments and service provision. In addition, data were collected also from studies providing differential estimates for, or focusing on population subgroups that are considered more at risk of influenza or influenza-related complications (e.g. children and the elderly and individuals with co-morbidities or complications).

A wide definition of influenza-related disease has been adopted, by including all studies where the target condition was labelled as laboratory confirmed influenza, influenza like illness (ILI); Parainfluenza Virus (PIV), acute respiratory infection (ARI), or respiratory syncytial virus (RSV). Conversely, studies estimating the costs of pandemic influenza, such as the 2009 *swine* flu pandemic, were not included in the review.

The primary outcomes of the present review are seasonal influenza-related direct health care costs. Studies could take either the healthcare sector perspective or a wider societal perspective. However, when a societal perspective was used, data were extracted for direct health-care costs only. When not directly provided, the cost per influenza-related episode was calculated by dividing the overall costs by the number of episodes reported. Conversely, studies were discarded whenever only aggregate estimates were reported, and conversion was not deemed possible.

When different estimates were provided (e.g. by age-group, or sex) an overall figure was calculated by using weighted averages (based on sample numbers), or using normal averages when the first was not possible. Also, when reported, a description of results by relevant subgroups in each study was provided.

Records were searched from January 2000 to December 2016 in electronic bibliographic databases including Medline (via Web of Science), Embase, Science Direct, the Cochrane Library and the Centre for Reviews and Disseminations of the University of York and Google scholar. The full search strategy was first defined on Web of Science, and then adapted to the other databases (S1 Table). Grey literature was retrieved through informal searches on Google. In addition, references from the included studies were hand-searched for completion.

After defining the search strategy, two reviewers independently performed title and abstract screening, full-text analysis and data extraction. At each stage, discrepancies were resolved by discussion with a third author. All records were imported in Endnote (ver. X6).

A standardized, pre-piloted form was used for data extraction including: study design; data sources; definition of the target disease; perspective and setting of the analysis; target patient groups and sample sizes. Cost data were extracted for the following items: ED and hospitalizations events; outpatient visits (with a distinction, if any, between GPs and specialists); prescription drugs and OTC drugs. Evidence on inappropriate influenza-related use of direct healthcare resources was also searched in the selected studies.

All costs were converted to 2015 PPP$ by using PPP conversion rates and GDP deflator series provided by the World Bank [11]. Finally, the PRISMA checklist [12] was used to monitor the reporting quality of the study (S1 Checklist)

## Results

**Fig 1:**
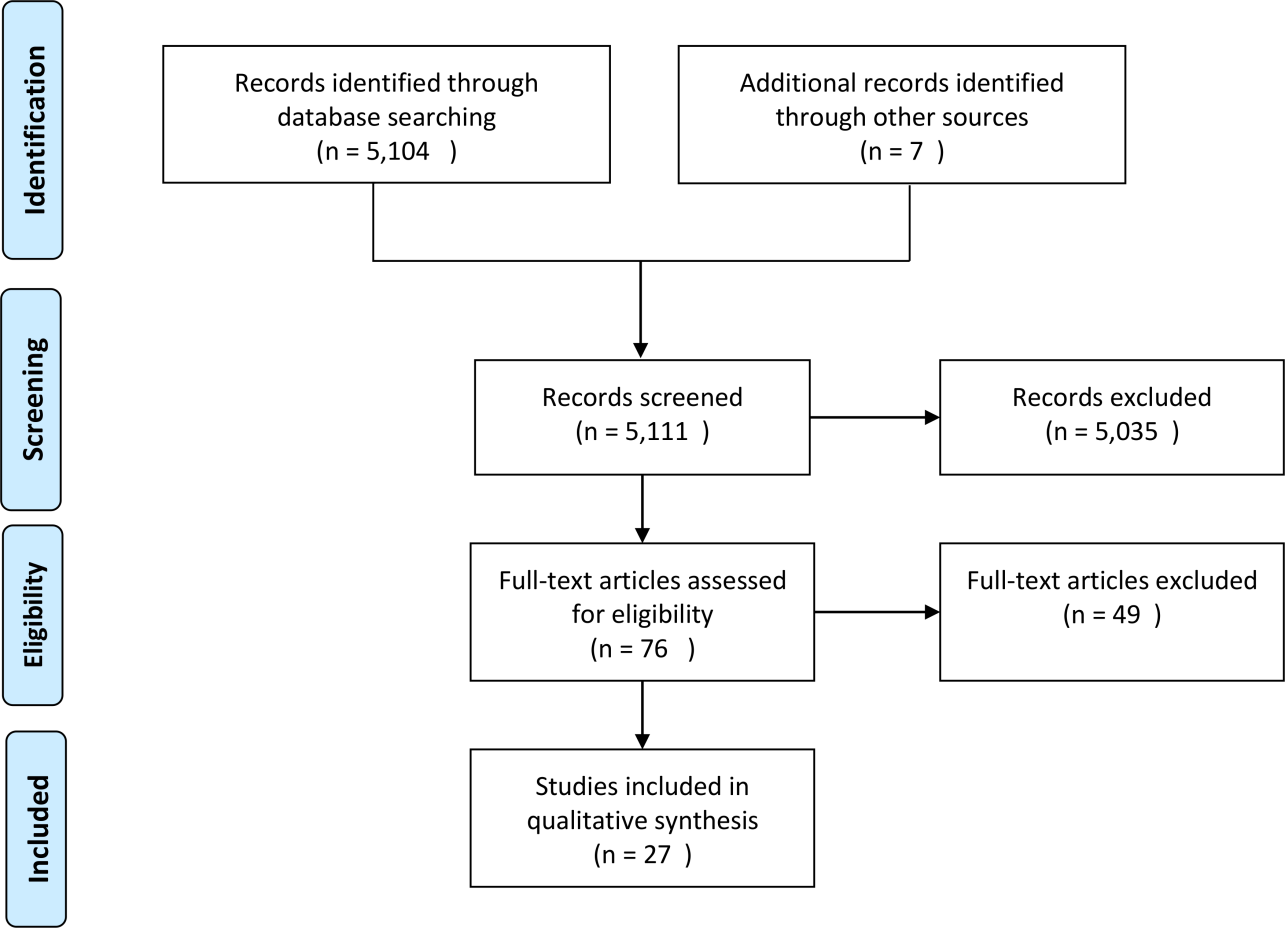
Study flow diagram.

After removing duplicates, the literature search identified 5,104 records through database searching, plus 7 other records from grey literature, hand searches of references, and informal searches on Google. After abstract and title screening, 76 records were analyzed full-text and 27 studies were finally included in the review (Fig 1). The full extraction template is provided as supporting material (S1 Dataset).

**Fig 2:**
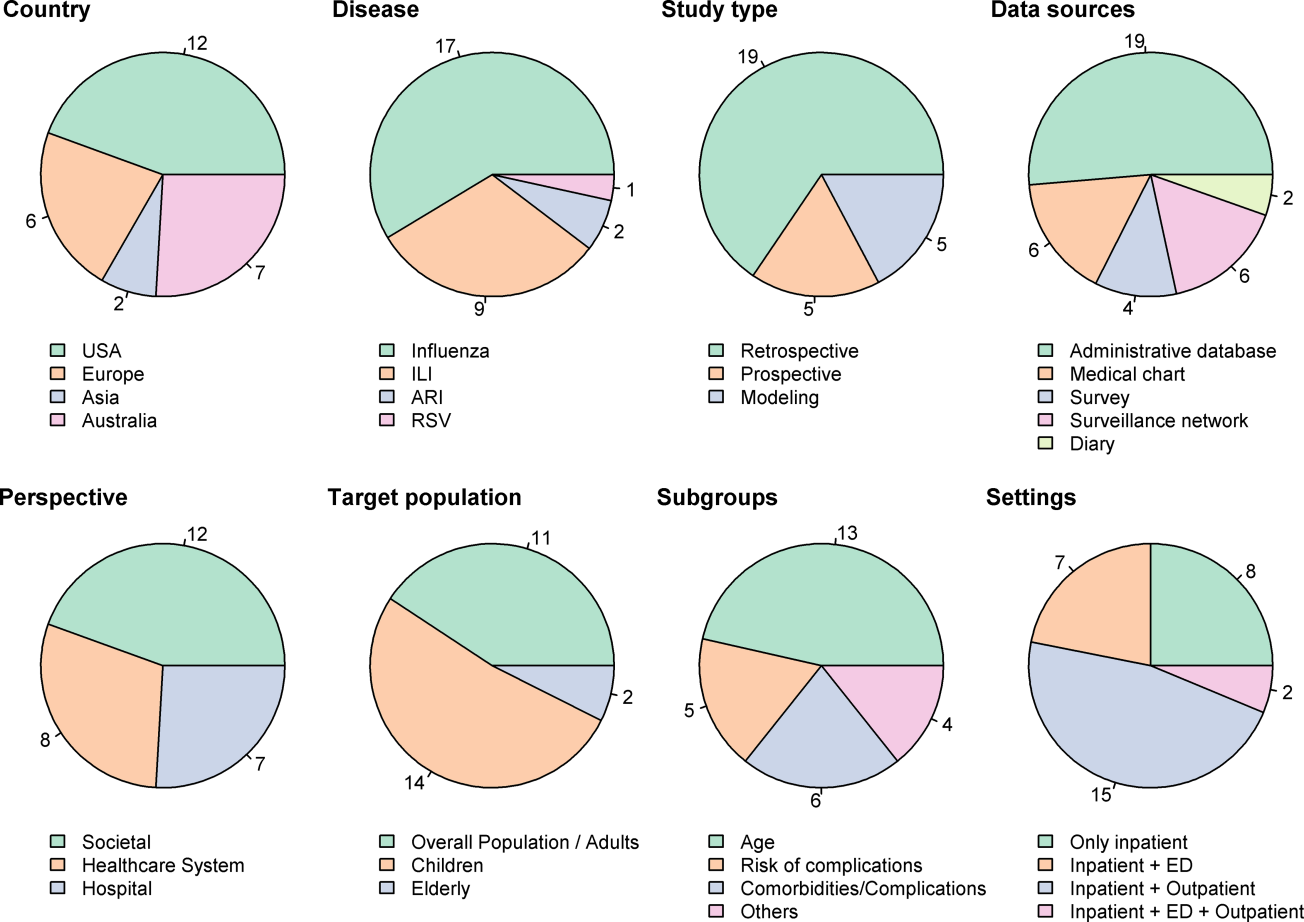
Descriptive characteristics of the studies.

Fig 2 and Table 1 illustrate the main characteristics of the studies included in the analysis. Of the included studies, 44% are based in the US, 22% and 26% in Europe and Australia respectively, and 8% in Asia. Retrospective analyses are predominant (66%) with most studies relying on administrative data. Societal perspective was adopted in 12 studies. The other studies took a health care system perspective (8 studies) or a hospital perspective (7 studies). Identification strategies of patients were mainly based on symptoms assessment, medical charts, confirmatory laboratory tests, or used international classification diseases codes (ICD-9-CM or ICD-10-CM code sets). Cases were mostly defined as influenza cases (59%), followed by ILI (31%), and to a minor extent ARI and RSV cases (7% and 3% respectively). Most of the studies focus on pediatric populations (52%), or the overall adult populations (41%), whereas elderly people were targeted in only 7% of the papers. More than 60% of the studies includes subgroups analyses by age (48%), co-morbidities and complications (22%), risk of complications (18%), and others (e.g. vaccinated *vs*. non-vaccinated patients) (15%).

**Table 1:** Descriptive characteristics of the studies.

Authors (year)	Country	Study type	Data sources	Disease (identification method)	Setting	Target population
Cox et al. (2000) [13]	USA	Retrospective	Administrative data	Influenza (ICD-9-CM)	Inpatient + ED	Overall population
Carrol et al. (2001) [14]	USA	Retrospective	Medical chart	ILI (symptoms based)	Inpatient + ED	Elderly
McBean et al. (2004) [15]	USA	Modeling	Administrative data, surveillance Network	Influenza and Pneumonia (ICD-9-CM)	Inpatient	Elderly
O’Grady et al. (2004) [16]	AUS	Prospective	Administrative data, diary	ILI (symptoms based)	Inpatient + ED+Outpatient	Children
Hall et al. (2005) [17]	USA	Retrospective	Administrative data	Influenza (ICD-9-CM)	Inpatient	Children
Ampofo et al. (2006) [18]	USA	Retrospective	Administrative data	Influenza (lab test)	Inpatient	Children
Keren et al. (2007) [19]	USA	Retrospective	Administrative data	Influenza (ICD-9-CM)	Inpatient	Children
Molinari et al. (2007)[2]	USA	Retrospective	Administrative data	Influenza and pneumonia (ICD-9-CM)	Inpatient + Outpatient	Overall population
Lambert et al. (2008) [20]	AUS	Retrospective	Administrative data, diary	ARI (laboratory test)	Inpatient + Outpatient	Children
Newall et al. (2008) [21]	AUS	Retrospective + modeling	Administrative data	Influenza, pneumonia and other respiratory illnesses (ICD 10)	Inpatient + Outpatient	Overall population
Hassan et al. (2009) [22]	USA	Retrospective + Modeling	Administrative data	Influenza (ICD-9-CM)	Inpatient	Children
Lester-Smith et al. (2009) [23]	AUS	Retrospective	Medical chart	Influenza (laboratory test)	Inpatient	Children
Fairbrother et al. (2010) [24]	USA	Retrospective	Surveillance Network	ARI (laboratory test)	Inpatient + ED	Children
Xue et al. (2010) [25]	NOR	Modeling	Administrative data, surveillance network	ILI (symptoms based)	Inpatient + Outpatient	Overall population
Esposito et al. (2011) [26]	ITA	Prospective	Administrative data, survey	ILI (medical chart and lab test)	Inpatient + Outpatient	Children
Ranmuthugala et al. (2011) [27]	AUS	Modeling	Administrative data, surveillance Network	RSV (laboratory test)	Inpatient	Children
Chiu et al. (2012) [28]	HKG	Prospective	Administrative data, medical chart	Influenza (laboratory test)	Inpatient	Children
Ortega-Sanchez et al. (2012) [29]	USA	Retrospective.	Administrative data, survey,medical chart	Influenza (laboratory test)	Inpatient + ED + Outpatient	Children
Karve et al. (2013) [30]	USA	Retrospective.	Administrative data	Influenza (ICD-9-CM)	Inpatient + ED + Outpatient	Overall population
Karve et al. (2013) [31]	USA	Retrospective.	Administrative data	Influenza (ICD-9-CM)	Inpatient + ED + Outpatient	Overall population
Suh et al. (2013) [32]	KOR	Retrospective.	Administrative data	Influenza (ICD-10-CM)	Inpatient + Outpatient	Overall population
Yin et al. (2013) [33]	AUS	Prospective.	Survey	ILI (laboratory test)	ED + Outpatient	Children
Enserink et al. (2014) [34]	NDL	Retrospective.	Survey	ILI (symptoms based)	Inpatient + Outpatient	Children
Silva et al. (2014) [35]	FRA	Prospective.	Surveillance Network	Influenza (laboratory test)	Inpatient + ED + Outpatient	Overall population
Pockett et al. (2015) [36]	GBR	Retrospective.	Medical chart	ILI (clinical chart)	Inpatient + Outpatient	Adult
Ehlken et al. (2015) [37]	DEU	Retrospective.	Medical chart	Influenza or ILI (ICD-10-CM)	Inpatient + Outpatient	Overall population
Haas et al. (2016) [38]	DEU	Retrospective.	Administrative data	Influenza or ILI (ICD-10-CM)	Inpatient + Outpatient	Overall population

Nine studies allow for a full estimate of the cost per ILI episode that include both inpatient and outpatient cases. The average cost per episode ranges from US$19 in Korea [32] to US$323 in Germany [39]. Particularly, the two studies that used laboratory tests to precisely identify influenza cases report a cost per episode of US$73 [26] and US$64 respectively [35] (Table 2).

**Table 2:** Costs reported in the included papers (US$ PPPs, 2015).

Study	Country	Costs per influenza-like episode^a^							Costs per hospitalized patient^b^	
		Inpatient	ED	Outpatient	GP	Prescription drugs	OTC	Total	Overall inpatient	% hospitalised
Cox et al. (2000) [13]	USA	– 1,107 – age <18y: 187 – age >65y: 1,504	– 198 – age <18y: 188 – age >65y: 264					1,305	– 4,526 – age <18y: 3,179 – age >65y: 5,042	24.4%
Carrol et al. (2001) [14]	USA	1,719	7			60		1,867	6,112	28.12%
McBean et al. (2004) [15]	USA								7,513	100%
O’Grady et al. (2004) [16]	AUS	42	1		10	4	13	87	2,805	2.54%
Hall et al. (2005) [17]	USA								– 17,459 – low risk: 3,944 to 11,663 – high risk: 11,791 to 49,807	100%
Ampofo et al. (2006) [18]	USA	2,218						2,218	– 8,027 – low risk. 5,244 to 7,354 – high risk: 9,122 to 14,162	27.6%
Keren et al. (2007) [19]	USA								– 16,209 – ICU: 49,014 – normal care: 8,659	100%
Molinari et al. (2007)[2]	USA			– low risk: 120 to 306 – high risk: 602 to 928^c^			4 (assumed in the model)		– low risk: 13,669 to 28,229 – high risk: 21,199 to 103,271	6.64%
Lambert et al. (2008) [20]	AUS				19 ^c^			27^c^	3,406	0.7%
Newall et al. (2008) [21]	AUS				41 ^c^				5,413	
Hassan et al. (2009) [22]	USA								– 3,539 – healthy: 3,525 – chronic cond. (no asthma): 9,839 – asthma: 4,390	100%
Lester-Smith et al. (2009) [23]	AUS								– 2,110 – PICU: 3,158 – normal care: 1,061	100%
Fairbrother et al. (2010) [24]	USA		593						6,254	48.21%
Xue et al. (2010) [25]	NOR	– 36 – no complications: 4 – pneumonia: 32		12 (includes also GP visits)		1	8 (assumed in the model)	74	– 4,816 – no complications: 3,247 – pneumonia: 8,091	0.01%
Esposito et al. (2011) [26]	ITA	– 26 – age <2y:49 – age 2-5y:29 – age>5y:13			39	4	2	– 73 – age <2y:83 – age 2-5y:75 – age>5y:59	– 3,564 – age <2y: 3,328 – age 2-5y: 3,807 – age>5y: 3,323	0.75%
Ranmuthugala et al. (2011) [27]	AUS								8,741	100%
Chiu et al. (2012) [28]	HKG								1,419	100%
Ortega-Sanchez et al. (2012) [29]	USA		– 801 – low risk: 874 – high risk: 568 ^c^				12 ^c^		– 3,402 – low risk: 3,663 – high risk: 2,413	
Karve et al. (2013) [30]	USA		– with complications: 876 – no complications: 764^d^	– with complications: 1,563 – no complications: 1,077 ^d^		– with complications: 857 – no complications: 572 ^d^		– with complications: 3,756 – no complications: 1,482 ^d^	– with complications:11,845 – no complications:7,758 ^d^	– with complications:7.1% – no complications:3.1%
Karve et al. (2013) [31]	USA	181	29	19	64	33	5 (defined as ancillary care)	321		from 1.31% to 2.03%
Suh et al. (2013) [32]	KOR	9		9		1		19	473	
Yin et al. (2013) [33]	AUS	0	40	3	55			169	0	0%
Enserink et al. (2014) [34]	NDL	16		25 (includes also GP visits)		7		46	3,217	
Silva et al. (2014) [35]	FRA	– 22 – age 0-4y:45 – age 5-14y:25	1		– 34 – age 0-4y:49 – age 5-14y:30	7		– 64 – age 0-4y:87 – age 5-14y:52	2,244	
Pockett et al. (2015) [36]	GBR	– 111 – low risk: 62 – high risk: 422		1	– 50 – low risk: 48 to 68 – high risk: 51 to 81	1		– 163 – low risk: 48 to 3,584 – high risk: 57 to 3,619	– 10,896 – low risk: 2,534 to 13,630 – high risk: 3,304 to 12,650	2%
Ehlken et al. (2015) [37]	DEU	– 17 – children: 18 – adults: 15		– 36 – children: 37 – adults: 34		– 13 – children: 14 – adults: 12		– 66 – children: 70 – adults: 62	– 2,332 – children: 2,274 – adults: 2,408	0.1%
Haas et al. (2016) [38]	DEU	75		235		9 overall (includes antibiotics, neuraminidase inhibitors. and others)	7 (includes analgesics, antitussives and others-nose spray)	315	6,469	0.4%

a When not reported directly, costs per episode were calculated by dividing the specific cost item by the total number of influenza cases (if available)

b When not reported directly, the hospitalization costs were calculated by dividing overall costs by the number of influenza-related hospitalizations

c Costs calculated per emergency and/or outpatient event (excluding inpatient cases)

d Costs per patient over a 12 months follow-up

(P)ICU: (paediatric) intensive care unit

On average, inpatient and outpatient services accounted for 43.5% (range 23–68%) and 44.5% (range 14–72%) of the total cost per episode, whereas incidence of drug costs was between 0.6% [36] and 19.7% [16] (Table 3).

**Table 3:** Impact of cost items on total costs per episode (%).

Author (year)	Country	Inpatient	ED	Outpatient	Prescription drugs	Over-the counter	Total Drugs	Total
O’Grady et al. (2004) [16]	AUS	60.0%	1.4%	14.3%	5.7%	18.6%	24.3%	100%
Xue et al. (2010) [25]	NOR	63.2%		21.1%	1.8%	14.0%	15.8%	100%
Esposito et al. (2011) [26]	ITA	36.6%		54.9%	5.6%	2.8%	8.5%	100%
Suh et al. (2013) [32]	KOR	47.4%		47.4%	5.3%		5.3%	100%
Enserink et al. (2014) [34]	NDL	33.3%		52.1%	14.6%		14.6%	100%
Silva et al. (2014) [35]	FRA	34.4%	1.6%	53.1%	10.9%		10.9%	100%
Ehlken et al. (2015) [37]	DEU	25.8%		54.5%	19.7%		19.7%	100%
Pockett et al. (2015) [36]	GBR	68.1%		31.3%	0.6%		0.6%	100%
Haas et al. (2016) [38]	DEU	23%		72.1%	2.1%	2.8%	4.9%	100%

The hospitalization costs over the total number of ILI cases (outpatient and inpatient cases) range between US$9 in Korea [32] and US$181 in the US [30], with differences partly explained by differential unit costs per hospitalization events and hospitalization rates. However, figures may also reflect differences in the estimation method (e.g. top down or bottom up approaches), the type of healthcare resources included and the monetary values attached to them.

As expected outpatient services considerably affect the overall cost per ILI episode, with most studies reporting a cost between US$1 and US$36. One notable exception is the work by Haas et al that estimated a total cost for outpatient visits of 259 million € over 1,2 millions of ILI cases (US$ 235 per episode) [39].

Evidence of impact of ED costs per ILI episode is less available since most of the studies reporting ED costs tend to adopt a narrower hospital perspective, whereas other studies with a wider perspective either disregarded ED costs or included them within inpatient services. However, the incidence on total cost per episode as reported in two studies was limited and about 1.5% [16,35].

Prescription and OTC drug costs were analysed in 12 and 13 studies respectively. The mean prescription drug cost per episode ranges from US$1 in UK [36], Norway [25], and Korea [32], to US$ 60 in the US [14], whereas in Europe, the maximum identified cost per episode was found in Germany (US$16) [38]. The expected OTC expenditure per episode ranges between US$2 in Italy [26] and US$13 in Australia and Germany [16,37], and their impact varied between 2.8% and 18.6% of total health care costs and between 13.2% and 88.9% of total drug costs. However, differences in OTC consumption is likely to be even more sensitive to estimation methods and data availability. Since OTC drugs are paid out-of-pocket, usage and overall costs have been either estimated through medical charts and patient diaries, or assumed by the authors. Antipyretics and antibiotics are the most-frequently reported drugs, integrated in a few studies by antiviral, analgesics, antitussives and nose spray drugs.

Thirteen studies stratify the population according to its age-structure. However, only few of these report estimates of the cost per episode. While children usually register higher costs, evidence for the elderly (> 65 years) is less conclusive, and heterogeneous across studies. In a US retrospective analysis using administrative data, treating elderly people was 35% more costly than the overall population [13], whereas a prospective study, using French surveillance data for 460 patients, reports no hospitalisations for people over 65 years, and an average cost per episode that is 20% less than the overall population [35].

Although based on a more limited number of studies, costs by patient risk-status are as expected: high-risk individuals require higher costs than low-risk ones, with inpatient services being the main cost driver. Only one study, using US administrative data, found lower costs for high-risk patients, although the difference was not statistically significant [29].

One study was found to provide full analysis of costs stratified by vaccination status [36]. According to this retrospective study using UK medical charts, vaccinated patients with complications experienced costs 4 to 5 times higher than unvaccinated and uncomplicated patients. This difference was justified by the circumstance that the vaccinated population includes older and riskier patients than the unvaccinated one.

Finally, co-morbidities and complications have an important impact on costs: a retrospective analysis on the costs of Influenza in US which relied on an administrative database of more than 50 thousands patients reports that on average complicated influenza is 2.5 times costlier than non-complicated influenza [30]. The difference is even higher in another study focused on ILI [36].

No studies were found that made explicit considerations on the appropriateness of pharmaceutical treatment for ILI episodes.

## Discussion and conclusions

Influenza and ILI impose a substantial burden on the health care sector and the society. The literature on the costs of this disease is substantial and focuses on the relevance of indirect and direct costs of the disease.

The present review has updated current knowledge by providing a picture of the overall cost per episode of ILI, the weight of each cost component, and the cost variability across patients’ sub-groups.

Cost estimates are different across countries, with an average cost per episode ranging between US$19 in Korea [32] to US$323 in Germany [39]. These differences may reflect country specific characteristics as well as other study-specific features including study design, the identification strategy of ILI cases, different study populations and types of costs included in the analysis.

Inpatient admissions absorb a considerable amount of healthcare resources. Therefore, potentially relevant sources of variation may be explained by different hospitalization rates, and the unit cost associated with each hospitalization event. The lowest cost per hospitalisation was reported in Korea (US$473) [32]; however, this cost is not fully comparable with other countries as the estimate includes only consultation, diagnostics and medication costs. In other countries, variations in hospitalization costs are still wide and range between US$ 1,419 in Hong Kong [40] and over US$ 7,000 in most US studies. In addition, in studies focusing on the overall ILI population, hospitalization rates varied in a range between near zero and 6%, that again may be attributed to within-study differences in the ILI population and other country-specific features.

The incidence of outpatient services on the cost per episode was similar to that of inpatient services, and equal to 44.5% on average (range 14–72%). Differences in costs of outpatient services reflect the observed variation in the number of outpatient visits per ILI episode. For example, Molinari et al estimated the probability of having an outpatient visit after a flu infection in the US to be in a range between 0.45 and 0.62 for low risk individuals, and 0.62 and 0.91 for high-risk individuals [2]. Conversely, Haas et al found an average number of outpatient visits in Germany equal to 6.6 visits per ILI episode [39], which ultimately affected the absolute and relative cost for outpatient services.

In addition, alternatively to virological confirmation via laboratory tests, many of the included studies identify influenza-like episodes using classification codes from medical claims (e.g. ICD-9-CM or ICD-10-CM), or a set of predefined symptoms and syndromes. These differences in the identification strategy may be a further source of variation in the estimated total and relative costs of care. For example, retrospective identification of ILI through administrative databases and ICD codes may include different patients populations compared to laboratory confirmed influenza cases (e.g. patients with bacterial pneumonia), that will ultimately affect resource consumption and costs. In addition, these different identification strategies reflect the fact that influenza cases are often not virologically confirmed in clinical practice. This aspect hamper any attempt to formulate considerations about the appropriateness of disease management strategies, especially regarding drug prescription.

None of the included studies has considered appropriateness in resource use (e.g. abuse of antibiotics). Despite cost of illness studies have ultimately a descriptive role, evidence on (in)appropriateness may be very useful for policy-makers. Indeed, there is a general belief that antibiotics are overprescribed in primary care. However, judgments on appropriateness of prescription are not easy and depend on clinical characteristics (e.g. presence of likely bacterial pathogens associated with influenza) [41]. A recent study estimated that antibiotic prescribing for ILI cases in UK ranges between 18% and 28% of total infections depending on the presence of comorbidities [42]. Similar proportions (25%) were found among subjects aged 0–65 at five military hospitals in the US [43]. Another UK study reported that, based on primary care prescribing guidelines, most antibiotics are prescribed for conditions that only sometimes require antibiotic treatment [44]. Among the included studies, the study by Silva et al [35] is the only one that provides details on drugs expenditures (antibiotics, antivirals and other drugs) in patients with confirmed Influenza B. The study reports that 46% of patients were prescribed antibiotics on average, while just 24% were given an antiviral.

While there is a paucity of studies specifically addressing inappropriate prescribing for ILI infections, inappropriateness may be inferred by the lower proportions of prescriptions that are generally registered when influenza virus rapid antigen tests are used [45,46].

Another disregarded topic is the role played by symptomatic medication (mainly OTC drugs): recent analyses from the grey literature stressed the importance of OTC drugs on healthcare expenditure, showing the potential economic burden for the US health care system, should OTC drugs not be available [47].

The present study has some limitations. First, we have not performed a quality assessment of the studies. Although envisaged in the PRISMA guidelines, it was considered that these types of costing studies, without a specific intervention, did not require a thorough assessment of the risk of bias. In addition, when not directly reported, data on the cost-per episode was calculated from other reported figures. Lastly the included papers were very different in terms of target population, definition of the disease, methods, data sources and outcomes, hampering the possibility of doing meaningful comparisons among them.

Despite these limitations, the paper provides an updated and complete analysis of the available evidence on Influenza and ILI health care costs.

## Supporting information

S1 Checklist PRISMA 2009 checklist. (DOC)

S1 Table Search strategy used for medline (via Web of Science). (DOCX)

S1 Dataset Full data from the included papers. (XLSX)
