# Supplementary material for: Health care costs of influenza-related episodes in high income countries: A systematic review
Source: PLoS One. 2018 Sep 7;13(9):e0202787. doi: 10.1371/journal.pone.0202787 (PMC6128484; doi:10.1371/journal.pone.0202787)
Supplement: S1 Table — (DOCX) [file pone.0202787.s002.docx]

Supplementary material 1

Search strategy used in Medline (via Web of science)

| #1 | TS=((cost NEAR/3 illness) OR burden OR (soc* NEAR/1 cost*) OR (medical NEAR/1 cost*) OR (healthcare NEAR/1 cost*) OR (health NEAR/1 care NEAR/1 cost*) OR (cost NEAR/1 influenza)) |
| --- | --- |
| #2 | TS:mapexp=(influenza) |
| #3 | #1 AND #2 |
